# In Situ Supramolecular Gel Formed by Cyclohexane Diamine with Aldehyde Derivative

**DOI:** 10.3390/polym14030400

**Published:** 2022-01-20

**Authors:** Jae-Hyeon Park, Min-Hye Kim, Moo-Lyong Seo, Ji-Ha Lee, Jong-Hwa Jung

**Affiliations:** 1 Department of Chemistry, Research Institute of Natural Sciences, Gyeongsang National University, Jinju 52828, Korea; parkjae@gnu.ac.kr (J.-H.P.); your1005@gnu.ac.kr (M.-H.K.); 2 Chemical Engineering Program, Graduate School of Advanced Science and Engineering, Hiroshima University, 1-4-1 Kagamiyama, Higashi-Hiroshima 739-8527, Japan

**Keywords:** LMOGs, supramolecular gel, urea reaction

## Abstract

Low-molecular-weight gels have great potential for use in a variety of fields, including petrochemicals, healthcare, and tissue engineering. These supramolecular gels are frequently metastable, implying that their properties are kinetically controlled to some extent. Here, we report on the in situ supramolecular gel formation by mixing 1,3-cyclohexane diamine (**1**) and isocyanate derivative (**2**) without any catalysis at room temperature in various organic solvents. A mixture of building blocks **1** and **2** in various organic solvents, dichloromethane, tetrahydrofuran, chloroform, toluene, and 1,4-dioxane, resulted in the stable formation of supramolecular gel at room temperature within 60–100 s. This gel formation was caused by the generation of urea moieties, which allows for the formation of intermolecular hydrogen-bonding interactions via reactions **1** and **2**. In situ supramolecular gels demonstrated a typical entangled fiber structure with a width of 600 nm and a length of several hundred μm. In addition, the supramolecular gels were thermally reversible by heating and cooling. The viscoelastic properties of supramolecular gels in strain and frequency sweets were enhanced by increasing the concentration of a mixed **1** and **2**. Furthermore, the supramolecular gels displayed a thixotropic effect, indicating a thermally reversible gel.

## 1. Introduction

**Figure 1:**
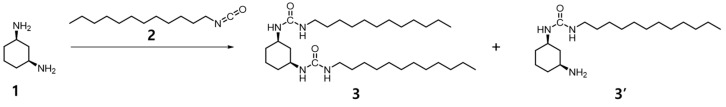
Reaction process to form in situ supramolecular gel by mixed compounds **1** and **2**.

Gels are easily recognized as soft materials with a wide range of applications in cosmetics, pharmaceuticals, and the food industry [1]. Gelled materials are colloidal systems made up of two coexisting phases: a liquid-like phase and a solid network, with the latter preventing the liquid from flowing in bulk [2]. Recently, there has been a greater emphasis placed on understanding the self-assembly and subsequent behavior of these materials [3]. The results from these studies are used to design carefully tailored gel materials for diverse applications from tissue engineering [4,5,6,7] to nanoscale electronics [8,9,10,11,12]. Generally, low-molecular-weight organogelators (LMOGs) are molecules that can form thermoreversible physical gels at concentrations less than 5% gelator/weight organic solvent [13,14,15,16,17,18]. Highly anisotropic 3D structures often form fibers during gelation, but they can also form ribbons, platelets, tubular structures, or cylinders [19,20,21,22]. Gelators can be classified into two categories according to the force driving their molecular aggregation: hydrogen-bond-based gelators [23,24,25,26] and non-hydrogen-bond-based gelators [27,28] (Figure 1). Aliphatic amide [29] or urea-coupled cyclohexane [30], peptides [31,32,33,34], and sugar-based derivatives with distinct helical structures [35,36,37] are typical examples of the former [38,39,40,41,42]. In contrast, cholesterol derivatives that aggregate due to crown moieties, π–π stacking, van der Waals forces, and/or solvophobic properties are a common example of the latter [43,44,45]. Among them, an intermolecular hydrogen-bonding interaction was utilized to prepare helical supramolecular gels. For instance, B. L. Feringa and Hanabusa groups reported that enantiomeric S, S-, or R, R-1,2-cyclohexane derivatives possessing two urea moieties formed organogels at low concentrations [46,47,48,49]. These cyclohexane-based gels showed the helical fiber structure with right or left helicity.

In situ supramolecular gelator formations have recently been demonstrated typically using two-component systems that form the gelator monomer mostly through covalent or noncovalent interactions [50,51,52,53,54,55,56,57,58,59,60]. There are several research studies of reaction based-gel systems where the mixing of different components leads to the formation of the actual gelator. For example, J. H. van Esch and R. Eelkema et al. [61,62,63,64] reported that catalytic action could be used to control the mechanical properties of in situ supramolecular hydrogels. In addition, they demonstrated that in situ catalysis of the formation of gelator molecules can accelerate the formation of supramolecular hydrogels, which drastically enhanced their resulting mechanical properties. D. J. Adams et al. [65] reported gel formation by exploiting dynamic covalent chemistry where the simple mixing of amine and aldehyde underwent imine bond formation reaction and thereby gelation occurs. They introduced the redox-responsive hydrogel system incorporating metal ions in gel media. However, only a few examples have been reported that allow for the formation of supramolecular gels in situ without the need for additional catalysis or heating. As a result, studying supramolecular gel formation in situ remains a challenge in supramolecular chemistry.

Herein, we report in situ supramolecular gel formed by mixed 1,3-cyclohexane diamine (**1**) and isocyanate derivative (**2**) as building blocks under various organic solvent system. Gel formation was related to the interaction based on the produced urea under organic solvent ranges, respectively. The formation of in situ supramolecular gel was caused by the formation of two urea moieties, which enabled the formation of the intermolecular hydrogen-bonding interaction as a result of the reaction of building blocks (**1**) and (**2**). In addition, the mechanical properties of the supramolecular gel were observed according to the self-assembly kinetics by controlling the concentration of (**1**) and (**2**) finely. The relationship between in situ supramolecular gel formation rate and strength according to concentration is discussed in detail. 

## 2. Materials and Methods

### 2.1. Reagents and Instruments

Precursors **1** and **2** were obtained from commercial suppliers (TCI, Sigma Aldrich, St. Louis, MO, USA) and used as the samples. The ^1^H NMR spectrum was obtained with a Bruker (ARX 300, Billerica, MA, USA) using a Bruker (ARX 300). A Thermo FT-IR Nicolet iS 10 (Thermo Fisher, Waltham, MA, USA) was used to measure the FT-IR spectra in ATR in the range of 400–4000 cm^−1^. Mass spectroscopy samples were analyzed on a Thermo Scientific LCQ Fleet mass spectrometer.

### 2.2. Preparation of Gels

First, heat and dissolve precursor **1** (9.5 μL, 8.75 μmol) in organic solvents such as dimethyl sulfoxide (DMSO), acetonitrile, dichloromethane, n-hexane, or toluene (1 mL). After adding the solution to precursor **2** (42.5 μL, 17.5 μmol), the reaction mixture was maintained for a set period to allow gel formation.

### 2.3. Preparation Method and Observation of SEM Samples

The freeze-dried gel sample was prepared from gel (3 wt %) by vacuum for 24 h. Then, the dried sample was mounted on aluminum stubs (12 mm diameter) with carbon tape, and it was coated with a thin layer of Pt. FE-SEM (Tescan, Brno, Czech Republic, S8000 field emission SEM) was used to obtain images using an accelerating voltage of 10–25 kV and an emission current of 10 mA. We used this instrument in the National Research Facilities & Equipment Center (2019R1A6C1010042).

### 2.4. Rheological Properties

The gels were loaded onto the rheometer plate according to the standard. Rheological properties were carried out by using AR-2000ex (TA Instruments Ltd., New Castle, DE, USA). A parallel plate with a diameter of 20 mm was used. The gap between the gel and the plate was set to 0.5 mm, and the experiments were carried out at 25 °C. Strain sweep tests were carried out with increasing amplitude oscillation from 0% to 1000% apparent strain on the shear. Frequency sweeps were carried out between 0.6283 and 62.83 rad s^−1^.

### 2.5. Synthesis of Compound ***3***

Compound **3** was prepared by a previously reported method [66]. Dodecylisocyanate (1.22 g, 5.7 mmol) was slowly added to a stirred solution of 1,3-cyclohexanediamine (0.3 g, 2.6 mmol) in toluene (30 mL). A gel-like precipitate was formed immediately. After stirring overnight, the crude product was collected by filtration as a white waxy solid. The product was purified by resuspending the waxy solid in dichloromethane (50 mL), stirring for 1 h, and collecting the product on a glass filter. This procedure was repeated when necessary. After drying at 60 °C under vacuum, the product was obtained as a white solid in 87% yield. The product was characterized by ^1^H NMR, FT-IR, and ESI-MS spectroscopy (Figures S1–S3). ^1^H NMR (500 MHz, DMSO-d_6_) δ 5.45 (s, 1H), 5.39 (d, *J* = 6.8 Hz, 1H), 3.39 (br, 2H), 3.06–2.98 (m, 4H), 2.04 (d, *J* = 12.2 Hz, 2H), 1.81 (d, *J* = 12.3 Hz, 2H), 1.70 (d, *J* = 14.1 Hz, 2H), 1.40 (s, 4H), 1.30 (d, *J* = 2.6 Hz, 32H). FT-IR (ATR): 3340, 3295, 2920, 2849, 1623, 1564, 1464, 1264, 1224, 722, 670, 636 cm^−1^. ESI-MS (*m*/*z*): Calculated for C_32_H_64_N_4_O_2_ [M + Na]^+^ 559.49, Found [M + NA]^+^ 559.50.

## 3. Results and Discussion

**Figure 2:**
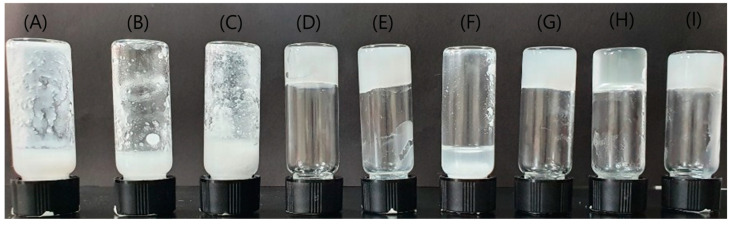
Photograph of supramolecular gels prepared by **1** and **2** in (**A**) DMF, (**B**) DMSO, (**C**) acetonitrile, (**D**) dichloromethane, (**E**) tetrahydrofuran, (**F**) n-hexane, (**G**) chloroform, (**H**) toluene, and (**I**) 1,4-dioxane.

Urea groups are excellent hydrogen-bond acceptors and donors, providing the noncovalent interactions required for self-assembly. The hydrophobic alkane core of urea-based gelator **3** is surrounded by two hydrogen-bonding urea groups. The alkyl chains were introduced to enhance the solubility of the building block (**2**) and prevent crystallization of the gelator (Figure 1). Importantly, the gelator can be synthesized in situ by reacting the 1,2-cyclohexane diamine building block **1** with two molecules of aldehyde **2**. Without any catalysis, a mixture of building blocks **1** and **2** in various organic solvents such as dichloromethane, tetrahydrofuran, chloroform, toluene, and 1,4-dioxane resulted in the stable formation of supramolecular gel at room temperature within 60–100 s (Figure 2).

To obtain evidence for the formation of urea groups, we used FT-IR spectroscopy to examine the FT-IR spectra of in situ supramolecular gels formed in various solvents (Figure S4). The medium vibration peak of -CN of 1-isocynatododecane (**2**) was observed at 2230 cm^−1^. In contrast, the medium vibration peak of -CN of a mixed sample consisting of building blocks **1** (1.0 Equiv.) and **2** (2.0 Equiv.) disappeared at 2265 cm^−1^ after 120 s. In contrast, the -C=O peak is generated at 1625 cm^−1^ (Figure S4C). This is clear evidence that urea was formed. Furthermore, the broad vibration peak of 1,3-cyclohexane diamine (**1**) was obtained at 3340 and 3275 cm^−1^, corresponding to -NH stretching, whereas a mixed sample of building blocks **1** and **2** obtained sharp peaks at 3300, 3338 cm^−1^, which were attributed to the generation of urea moieties by the reaction of **1** and **2**. Thus, the urea moieties of gelator **3** acted as a driving force in the formation of in situ supramolecular gel. To analyze the quantitative amount of desired product **3** in situ gel obtained by a mixing **1** and **2**, we prepared compound **3** and measured ^1^H NMR spectrum in DMSO-d_6_ at 100 °C to prevent gel formation. As shown in Figure S5C, two different NH protons attached to the cyclohexane, and the alkyl chain groups appeared at 5.44 and 5.37 ppm, respectively. In addition, ^1^H NMR spectrum of the xerogel sample obtained from a mixed of **1** (1.0 equiv.) and **2** (2.0 equiv.) in toluene was observed in DMSO-d_6_ at 100 °C (Figure S5D). As expected, two different NH protons of urea moieties appeared at 5.44 and 5.37 ppm, respectively, as obtained from pre-synthesized **3**. In particular, the ratio of NH protons and CH_3_ of alkyl chain group was 2:3 by the integral ratio, indicating that the xerogel sample consists of compound **3**. Based on NMR and FT observations, in situ supramolecular gel by a mixture of **1** (1.0 equiv.) and **2** (2.0 equiv.) was produced ca. 98% of desired product **3**. In contrast, about 2% of monourea (**3’**) as a minar product would exist in situ gel. We also obtained the evidence of the generation of gelator **3** by electrospray ionization-mass spectroscopy (ESI-MS) (Figure S7). A sample was observed by ESI-MS after the mixed sample of **1** and **2** was kept at room temperature without stirring. The main peak was found at *m*/*z* = 559.4921, corresponding to [**3+H**]^+^. The corresponding peaks for **1** and **2** were not seen. These findings suggest that gelator **3** generated rapidly by mixing **1** and **2** at room temperature within 120 s. Additionally, temperature-dependent proton NMR spectra were observed from 25 to 100 °C in DMSO-d_8_ (Figure S8). When the temperature was raised, the -NH protons attached to the urea moieties of **3** gradually shifted to a high field, which was attributed to the dissociation of the intermolecular hydrogen-bonding interaction between the urea moieties of **3**. This is clear evidence that the urea moieties of **3** acted as a driving force to form in situ gel. The supramolecular gel became a transparent solution by heating (110 °C), and then, the solution was returned to gel at room temperature again (Figure S9), which means that the supramolecular gel was thermally reversible. 

**Figure 3:**
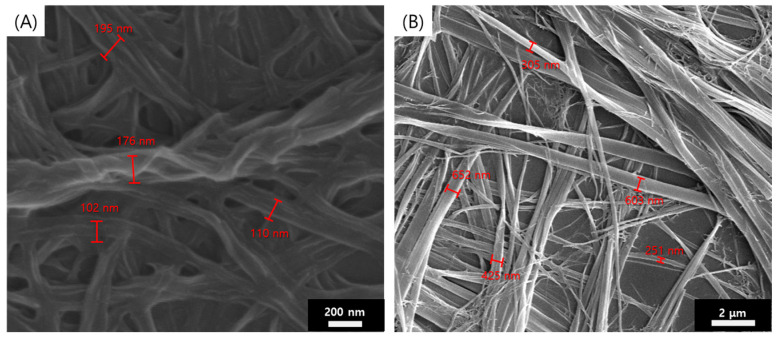
SEM images of in situ supramolecular gels prepared by **1** (3 wt %) and **2** (3 wt %) in (**A**) toluene and (**B**) n-hexane.

To examine the morphology of a supramolecular gel formed by a mixture of **1** and **2**, we used SEM to examine samples prepared in various solvents (Figure 3 and Figure S10). The supramolecular gel showed typical entangled fiber structures with 100–700 nm of width and several hundred μm of length. There were no significant morphology changes in supramolecular gels formed in various solvents. 

**Figure 4:**
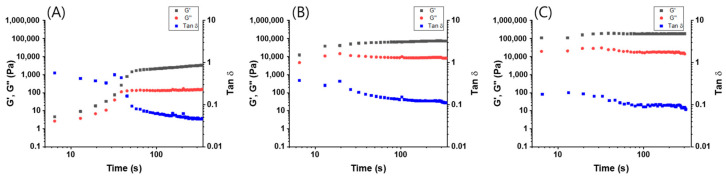
Time sweep (frequency = 0.6283 rad s^−1^) of gels obtained from (**A**) **1** (1 wt %) + **2** (2 equiv.), (**B**) **1** (3 wt %) + **2** (2 equiv.) and (**C**) **1** (5 wt %) + **2** (2 equiv.) in toluene.

Finally, we investigated the mechanical properties (G′ and G″) of the supramolecular gels in the linear viscoelastic region. The time-dependent storage (G′) and loss (G″) moduli of the supramolecular gel prepared at three different concentrations of **1** + **2** in toluene were measured (Figure 4). When mixing **1** and **2**, the G′ and G″ values of supramolecular gel (5.0 wt %) were immediately increased, and then, these values were maintained at their maximum. On the other hand, the G′ and G″ values of supramolecular gel (1.0 wt %) reached a maximum after 50 s. The G′ value of supramolecular gel prepared at 5.0 wt % was ca. 10-fold higher than that the supramolecular gel prepared at 1.0 wt %, which was ascribed to the formation of a three-dimensional entangled fiber structure in higher concentration. The gelation time decreased as the concentration of **1** + **2** in toluene increased, and the stiffness of the supramolecular gel increased and maintained the solid-like behavior.

**Figure 5:**
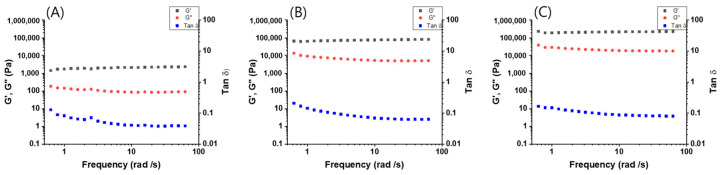
Frequency sweep of gels obtained from (**A**) **1** (1 wt %) + **2** (2 equiv.), (**B**) **1** (3 wt %) + **2** (2 equiv.) and (**C**) **1** (5 wt %) + **2** (2 equiv.) in toluene.

Furthermore, strain sweeps of supramolecular gels prepared at various concentrations of **1** + **2** in toluene were observed (Figure S11). As expected, the G′ and G″ values of the supramolecular gel prepared at 5.0 wt % were ca. 100-fold higher than that the supramolecular gel prepared at 1.0 wt %. The G′ and G″ values of the supramolecular gel prepared at 5.0 wt % showed a consistent tendency to those of the supramolecular gel prepared at 3.0 wt %. At 100% of strain, all three conditions of supramolecular gel (1 wt %, 3 wt %, and 5 wt %) reversed to G′/G″ < 1. These findings indicate that supramolecular gels behave as liquids at 100% of strain. However, as the **1** + **2** concentration increased, the collapse started with G′/G″ < 1 at low strain. In the case of 1 wt % of supramolecular gel, the values of G′ and G″ showed the lowest value, but the gel breakdown point was required at the greatest strain; therefore, the gel formed at 1 wt % showed the highest strength. Similar behavior was observed in the strain percentage change from 0.1% to 1000%, except that the strength of supramolecular gels increased with increasing gel concentration. There is no significant change at 0.1–2% of the stain. After 2% of strain, it showed a decrease. Frequency sweeps were performed from 0.6283 to 62.83 rad s^−1^, where significant changes in the G′ and G″ values were observed by an increase of concentration (**1** + **2**) (Figure 5). When the concentration of **1** + **2** was increased from 1 wt % to 3 wt % and 5 wt %, the G′ and G″ values increased 20-fold and 100-fold, respectively, when compared to 1 wt % of supramolecular gel. With increasing the concentration of gelators, the supramolecular gel leads with high strength. The G′ and G″ values of supramolecular gels were kept constant despite a frequency change of 0.6283 to 62.83 rad s^−1^. As the reference experiment, we also measured the mechanical properties of gel obtained from pre-synthesized **3**. The time-dependent storage (G′) and loss (G″) moduli of the supramolecular gel prepared from pre-synthesized **3** in toluene were measured (Figure S12). As observed for in situ supramolecular gel, the G′ and G″ values of gel (1.0 wt %) obtained from **3** reached a maximum after 50 s. The G′ and G″ values were almost same to those for in situ supramolecular gel (1 wt %). Furthermore, no significant differences between gel obtained from pre-synthesized **3** (1 wt %) and in situ supramolecular gel prepared from a mixed **1** (1 wt %, 1.0 equiv.) and **2** (2.0 equiv.) in frequency and strain sweets were observed. These findings indicate that a mixing **1** and **2** in toluene was converted to compound **3** in the gel formation. 

## 4. Conclusions

In this work, we have demonstrated the formation of supramolecular gel formed by mixing **1** and **2** at room temperature without catalysis. The urea groups formed by the reaction of building blocks **1** and **2** acted as a driving force for in situ supramolecular gelations. The supramolecular gels were also thermally reversible by heating and cooling. The morphology of supramolecular gels showed three-dimensional entangled network fiber structures. Furthermore, increasing the concentration of a mixed **1** and **2** increased the mechanical properties (G′ and G″) of the supramolecular gels prepared in toluene. The supramolecular gel prepared at 5.0 wt % has the greatest gel strength. Moreover, the supramolecular gels demonstrated a thixotropic effect, indicating a thermally reversible gel. Thus, we believe that the further development of in situ supramolecular gels formed at room temperature by mixing amine, hydrazine, and aldehyde derivatives will provide materials for bio-related applications by integrating functional derivatives.

## Data Availability

The data presented in this study are available in article.

## Supplementary Materials

The following supporting information can be downloaded at: https://www.mdpi.com/article/10.3390/polym14030400/s1, Figure S1: ^1^H NMR (500 MHz) spectrum of pre-synthesized compound **3** in DMSO-d_6_ at 100 °C; Figure S2: IR spectrum of pre-synthesized compound **3**; Figure S3: ESI MS spectrum of pre-synthesized compound **3** (1 × 10^−5^ M) in toluene; Figure S4: IR spectra of (A) **1**, (B) **2**, (C) supramolecular gels prepared by **1** and **2** in toluene, Figure S5 ^1^H NMR spectra of (A) **1,** (B) **2**, and (C) pre-synthesized **3** at 100 °C, and (D) xerogel in DMSO-d_6_ at 100 °C; the xerogel sample was prepared by a mixed **1** (3 wt %) + **2** (2 equivalent) in toluene; Figure S6: IR spectra of in situ gel prepared by **1** and **2** after mixing (A) 60 s, (B) 120 s, (C) 180 s, in toluene; Figure S7: ESI mass spectrum of diluted solution (1 × 10^−5^ M) prepared from in situ gel with a mixed **1** (3 wt %) + **2** (3 wt %) in toluene; Figure S8: Temperature-dependent ^1^H NMR spectra of xerogel sample in DMSO-d_6_; the sample was prepared by a mixed **1** (3 wt %) + **2** (2 equivalent) in toluene; Figure S9: Photograph of **1** (0.005 mM) with **2** (0.01 mM) (A) at room temperature, (B) at 110 °C, (C), after room temperature; Figure S10: SEM images of in situ supramolecular gels prepared by **1** and **2** in (A) chloroform and (B) tetrahydrofuran; Figure S11: Time sweep (frequency = 0.6283 rad s^−1^) of gels obtained from (A) **1** (1 wt %) + **2** (2 equivalent), (B) **1** (3 wt %) + **2** (2 equivalent), (C) **1** (5 wt %) + **2** (2 equivalent) in toluene; Figure S12: (A) Time- (frequency = 0.6283 rad s^−1^), (B) frequency-, and (C) strain-sweep at 0.1–1000% of gel obtained from (A) pre-synthesized compound **3** (1 wt %) in toluene.
