# Supplementary material for: In Situ Supramolecular Gel Formed by Cyclohexane Diamine with Aldehyde Derivative
Source: Polymers (Basel). 2022 Jan 20;14(3):400. doi: 10.3390/polym14030400 (PMC8840383; doi:10.3390/polym14030400)
Supplement: Supplementary file 1 [file polymers-14-00400-s001.zip › polymers-1532952-supplementary.pdf]

Supplementary Material

In Situ Supramolecular Gel Formed by Cyclohexane Diamine with Aldehyde Derivative

Jaehyeon Park [1,†], Minhye Kim [1,†], Moo Lyong Seo [1,*], Ji Ha Lee [2,*] and Jong Hwa Jung [1,*]

1 Department of Chemistry and Research Institute of Natural Sciences, Gyeongsang National University, Jinju 52828, Korea; parkjae@gnu.ac.kr (J.P.); your1005@gnu.ac.kr (M.K.)
2 Chemical Engineering Program, Graduate School of Advanced Science and Engineering, Hiroshima University, 1-4-1 Kagamiyama, Higashi-Hiroshima 739-8527, Japan
* Correspondence: mlseo@gnu.ac.kr (M.L.S.); leejiha@hiroshima-u.ac.jp (J.H.L.); jonghwa@gnu.ac.kr (J.H.J.)
† These authors contributed equally in this work.

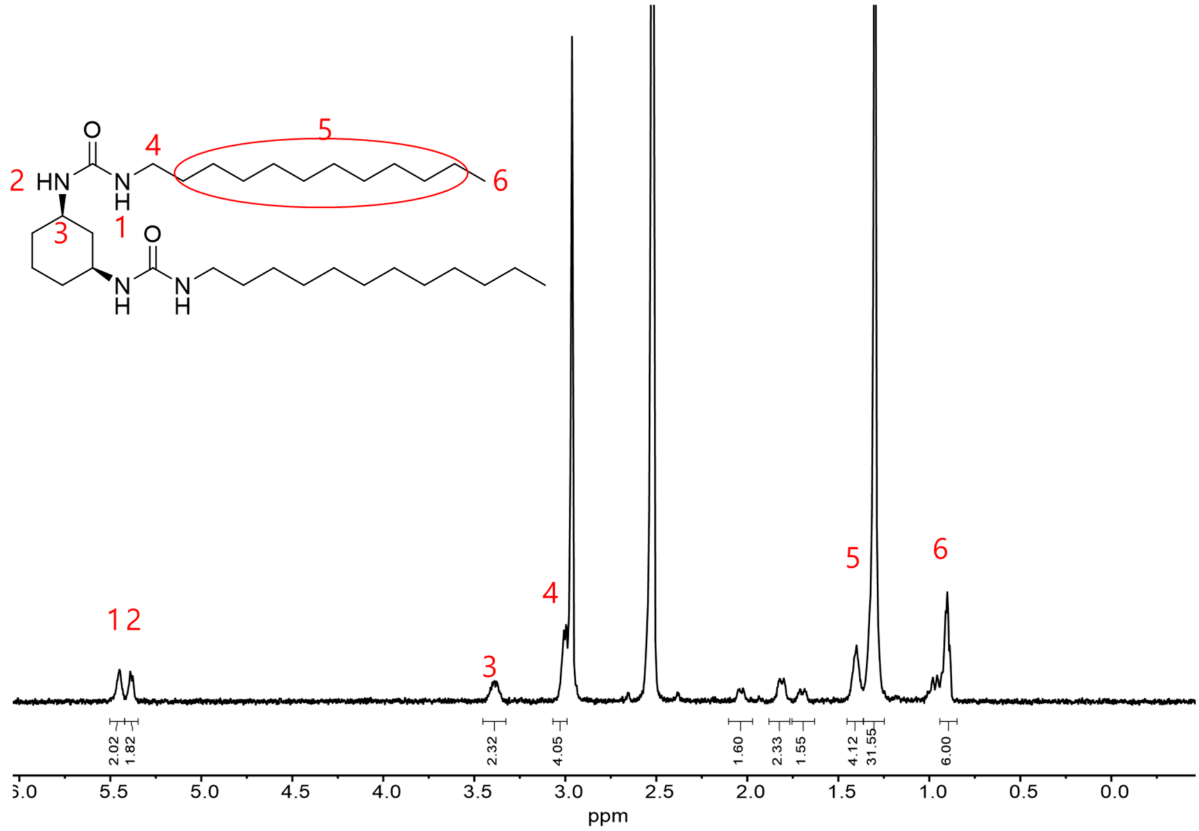

Figure S1. ^{1}H NMR (500 MHz) spectrum of pre-synthesized compound **3** in DMSO-d_6 at 100 °C.

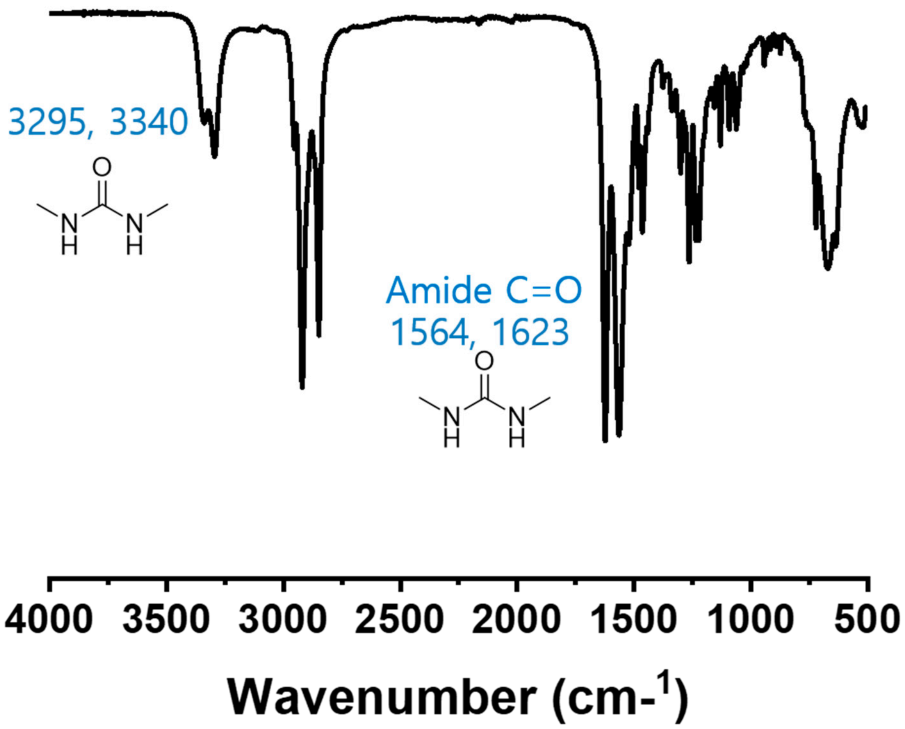

Figure S2. IR spectrum of pre-synthesized compound **3**.

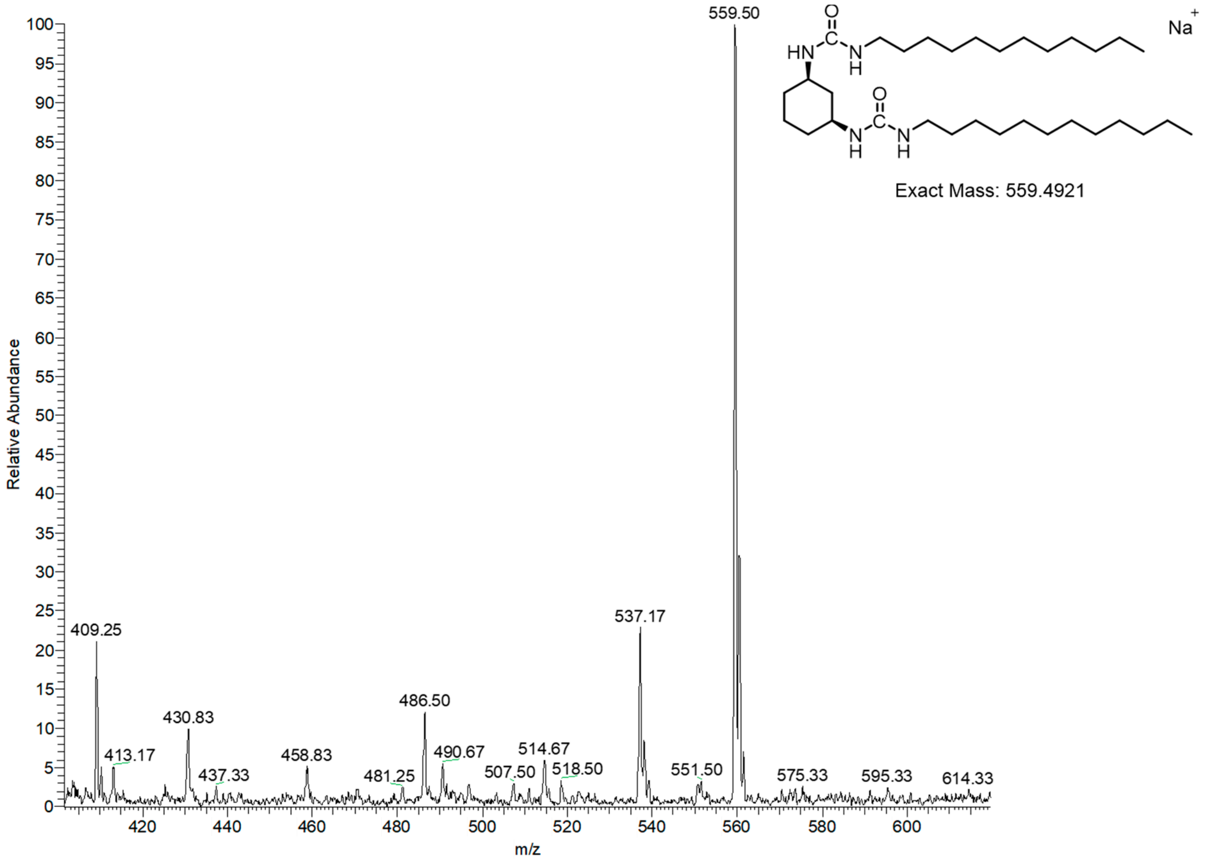

Figure S3. ESI mass spectrum of pre-synthesized compound **3** (1 x 10^{-5} M) in toluene.

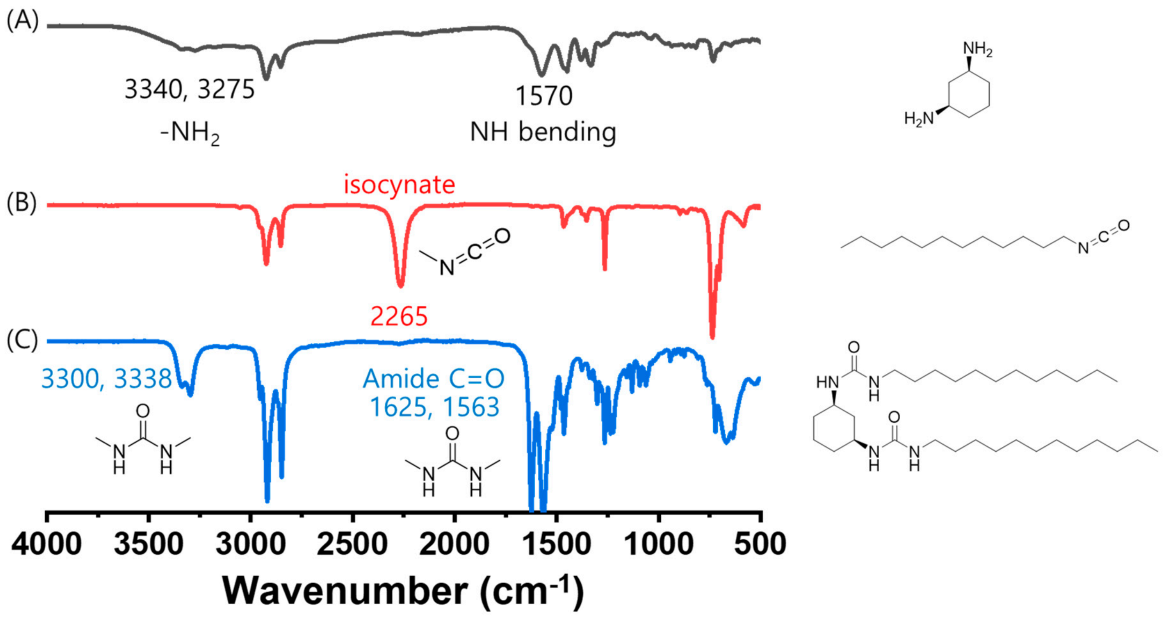

Figure S4. IR spectra of (A) **1**, (B) **2**, and (C) supramolecular gels prepared by **1** and **2** in toluene.

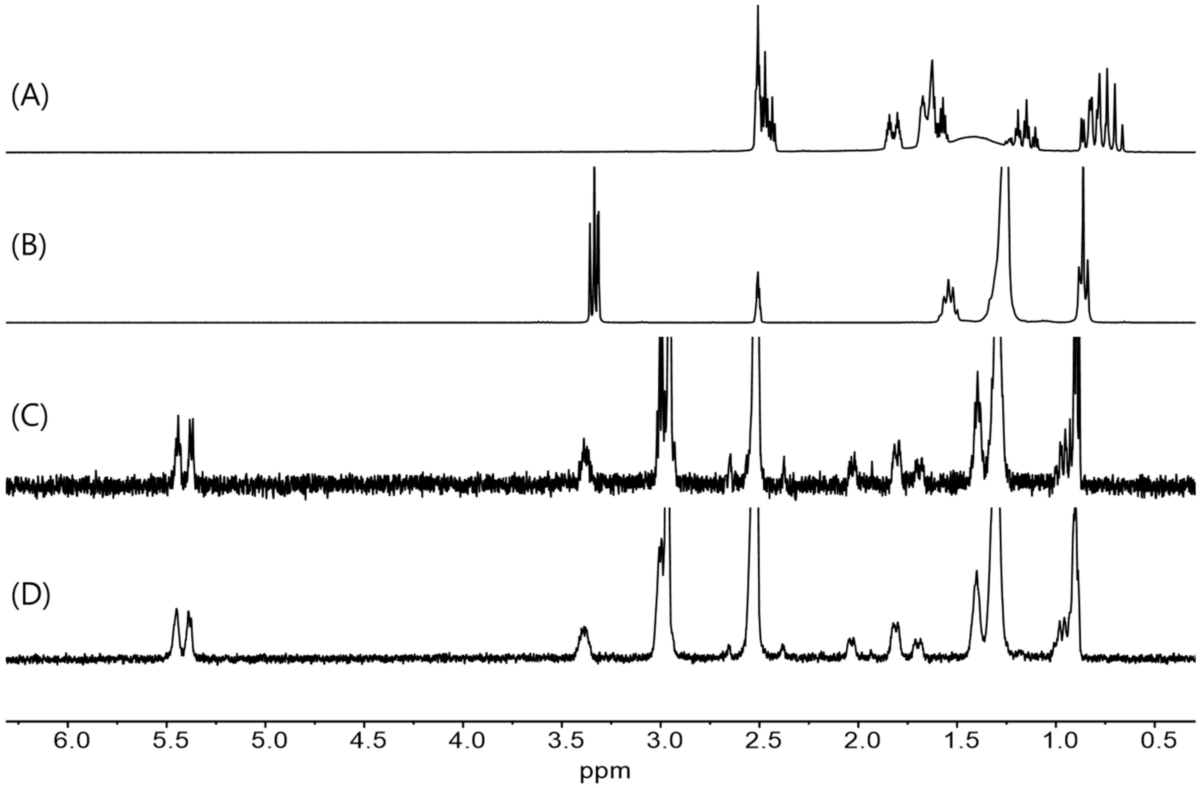

Figure S5. ^{1}H NMR spectra of (A) **1,** (B) **2**, (C) pre-synthesized compound **3** and (D) xerogel in DMSO-d_6. The samples (C and D) were measured at 100 °C. After preparation in situ supramolecular gel by a mixed **1** (1.0 equiv.) and **2** (2.0 equiv.) in toluene after reaction for 2 min, the xerogel sample was obtained by freeze dry.

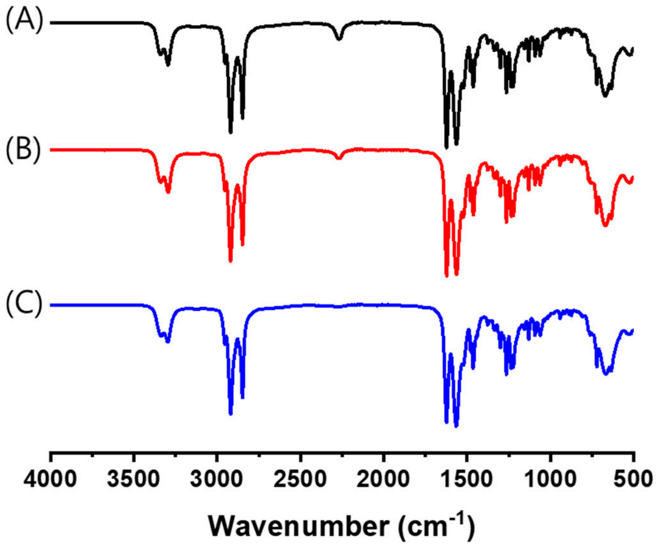

Figure S6. IR spectra of in situ gel prepared by **1** (1.0 equiv.) and **2** (2.0 equiv.) after mixing (A) 60 second, (B) 120 second, (C) 180 second in toluene.

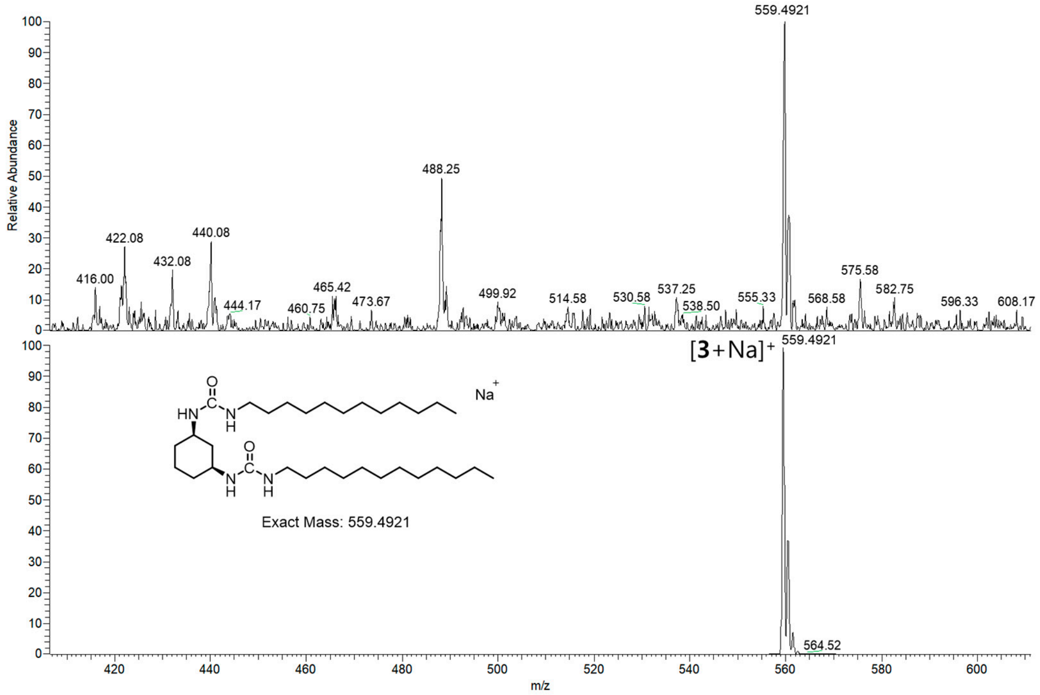

Figure S7. ESI mass spectrum of diluted solution (1 x 10-5 M) prepared from in situ gel with a mixed **1** **(**3 wt%) + **2** (3 wt%) in toluene.

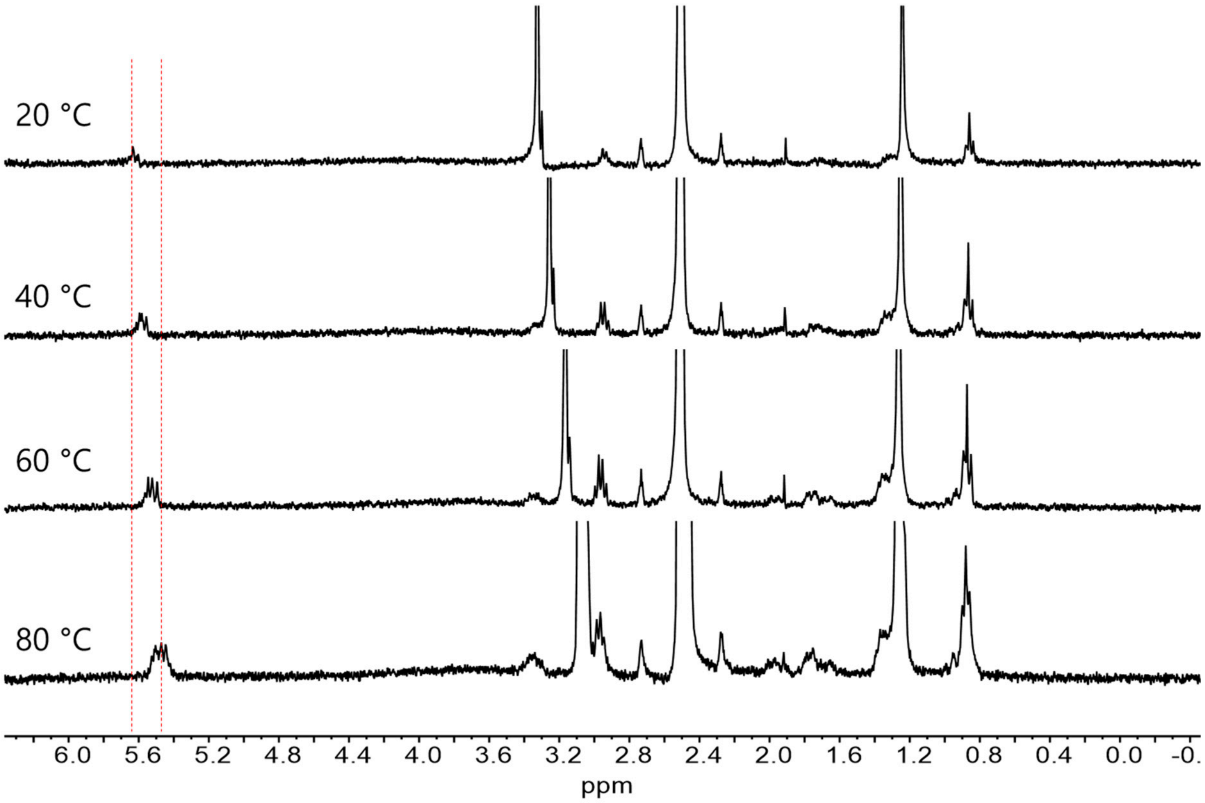

Figure S8. Temperature-dependent ^{1}H NMR spectra of xerogel sample in DMSO-d$_6$. The sample was prepared by a mixed **1** (3 wt%) + **2** (2 equivalent) for 2 min in toluene.

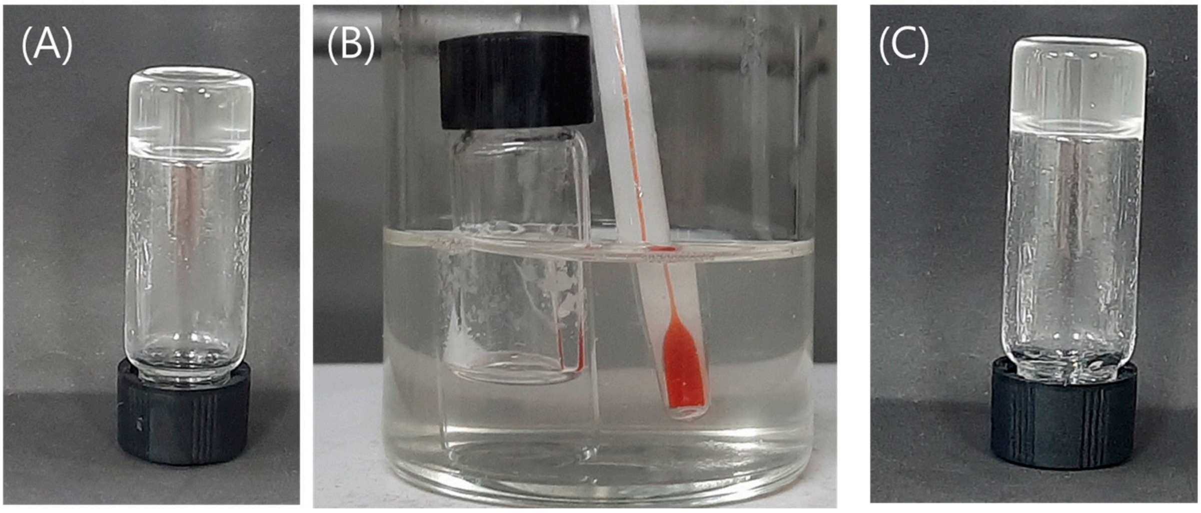

Figure S9. Photograph of **1** (0.005 mM) with **2** (0.01 mM) in toluene (A) at room temperature, (B) at 110 °C, (C), and after room temperature.

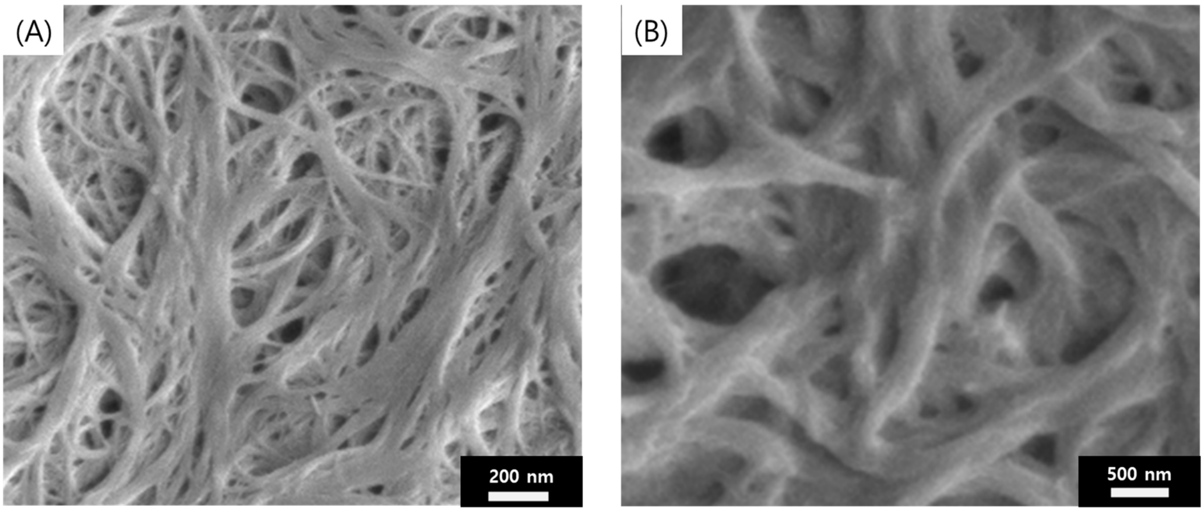

Figure S10. SEM images of in situ supramolecular gels prepared by **1** (3 wt%) and **2** (wt%) in (A) chloroform and (B) tetrahydrofuran.

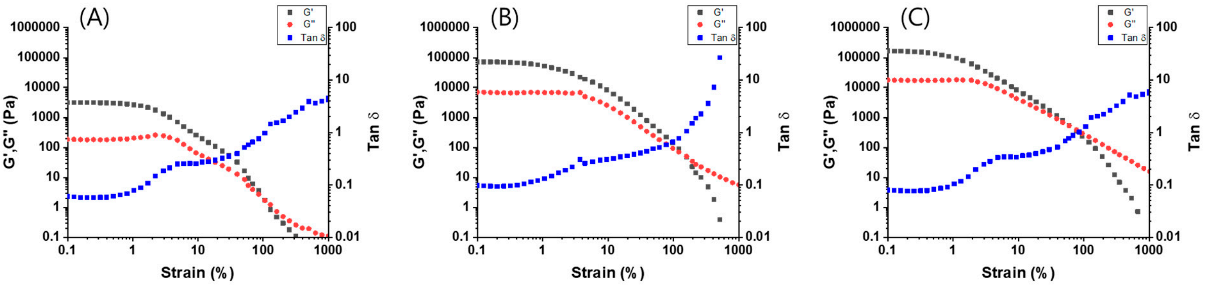

Figure S11. Strain sweep at 0.1%–1000% (frequency = 0.6283 rads^{-1}) of gels obtained from (A) **1** (1 wt%) with **2** (2 equiv.), (B) **1** (3 wt%) with **2** (2 equiv.) and (C) **1** (5 wt%) with **2** (2 equiv.) in toluene.

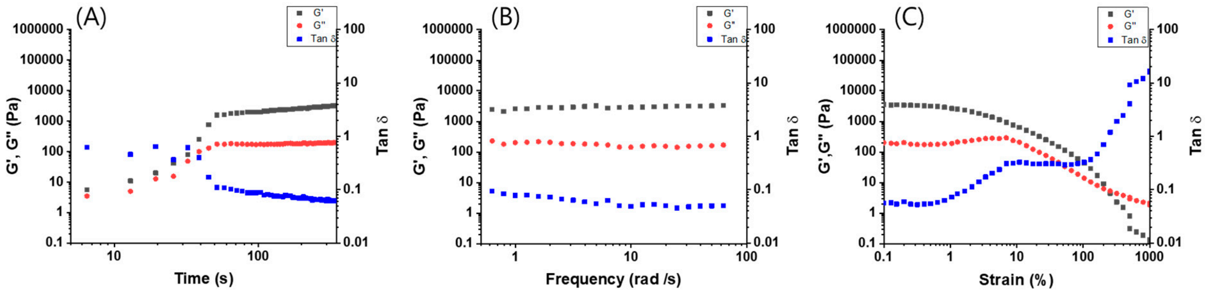

Figure S12. (A) Time- (frequency = 0.6283 rads^{-1}), (B) frequency-, and (C) strain-sweep at 0.1%–1000% of gel obtained from (A) pre-synthesized compound **3** (1 wt%) in toluene.
